# Effective Immobilization of *Agrobacterium* sp. IFO 13140 Cells in Loofa Sponge for Curdlan Biosynthesis

**DOI:** 10.3390/molecules20057957

**Published:** 2015-05-04

**Authors:** Camila Ortiz Martinez, Suelen Pereira Ruiz, Marcela Tiemi Nogueira, Evandro Bona, Márcia Portilho, Graciette Matioli

**Affiliations:** 1 Postgraduate Program in Food Science, State University of Maringá (UEM), Av. Colombo, 5790, Maringá-PR 87020-900, Brazil; E-Mails: camila_ortizmartinez@yahoo.com.br (C.O.M.); suelen_ruiz@hotmail.com (S.P.R.); 2 Pharmacy Department, State University of Maringá (UEM), Av. Colombo, 5790, Maringá-PR 87020-900, Brazil; E-Mails: marcelatiemi@hotmail.com (M.T.N.); mportilho@uem.br (M.P.); 3 Food Engineering Department, Federal Technological University of Paraná (UTFPR), Via Rosalina Maria dos Santos, 1233, Campo Mourão-PR 87301-899, Brazil; E-Mail: ebona@utfpr.edu.br

**Keywords:** curdlan, *Agrobacterium* sp., immobilization, mixed-level design, loofa sponge

## Abstract

Curdlan production by *Agrobacterium* sp. IFO13140 immobilized on loofa sponge, alginate and loofa sponge with alginate was investigated. There was no statistically-significant difference in curdlan production when the microorganism was immobilized in different matrices. The loofa sponge was chosen because of its practical application and economy and because it provides a high stability through its continued use. The best conditions for immobilization on loofa sponge were 50 mg of cell, 200 rpm and 72 h of incubation, which provided a curdlan production 1.50-times higher than that obtained by free cells. The higher volumetric productivity was achieved by immobilized cells (0.09 g/L/h) at 150 rpm. The operating stability was evaluated, and until the fourth cycle, immobilized cells retained 87.40% of the production of the first cycle. The immobilized cells remained active after 300 days of storage at 4 °C. The results of this study demonstrate success in immobilizing cells for curdlan biosynthesis, making the process potentially suitable for industrial scale-up. Additional studies may show a possible contribution to the reduction of operating costs.

## 1. Introduction

Curdlan is a neutral linear exopolysaccharide that is composed exclusively of more than 12,000 subunits of glucose linked by β-(1,3)-glycosidic bonds, with an average molecular weight of 74,000 Da. The number of glucose subunits and molecular weight vary with the characteristics of the producing strain. Curdlan was initially biosynthesized in conditions of nitrogen limitation using *Alcaligenes faecalis* variety myxogenes, now identified as *Agrobacterium biovar* 1 [1,2]. Until now, only bacteria belonging to the *Rhizobium*, *Alcaligenes*, *Agrobacterium*, *Cellulomonas* and *Bacillus* species have been reported to produce these biopolymers [3,4,5,6,7].

The transcriptome profiling of *Agrobacterium* sp. ATCC 31749 revealed a conserved regulatory mechanisms of curdlan biosynthesis. The analysis revealed around 100-fold upregulation of the curdlan synthesis operon upon transition to nitrogen exhaustion, thus setting the prominent role that transcriptional regulation exerts on exopolysaccharide synthesis. Along with genetic, biochemical and physiological analysis, the authors revealed a multifaceted network of regulation for curdlan biosynthesis, including acidocalcisomes, RpoN (Sigma factor 54 )-independent nitrogen regulation, c-di-GMP (bis-(3',5')-cyclic-dimeric-guanosine monophosphate), polyP (Polyphosphate) metabolism and the stringent response. The stringent response signal, pppGpp (Guanosine pentaphosphate), appeared to be essential for transcriptional activation of curdlan biosynthesis [8].

Curdlan production is of great interest due to its unique rheological and thermal gelling properties. When suspended in water, curdlan can form two different types of gels by temperature adjustment: a thermo-reversible, weak gel obtained by heating at 60 °C and subsequent cooling and a strong, thermo-irreversible gel obtained at temperatures greater than 80 °C [9]. The typical properties of curdlan have prompted its use in texture improvement, water retention and the thermal stability of diverse foods. Indeed, curdlan has frequently been reported as useful additive for several food products [10].

High potential for curdlan use has also been found in the pharmaceutical industry, because of its potent biological activities. The biopolymer acts as an immune stimulator against bacterial and viral infections, and it has been found to exhibit high anti-acquired immunodeficiency syndrome (AIDS) virus activity [11]. Curdlan sulfate exhibits strong inhibitory action on blood coagulation and can thus be used for the treatment of thrombosis [12]. Research also reported its application in the production of biodegradable plastics for medical applications [13] and its use as a drug delivery carrier for the sustained release of drugs [14].

The demand for curdlan applications is increasing and requires a highly-efficient production process. Mutant strains with increased curdlan production capacity have been identified [15]. The effect of pH on curdlan production has been evaluated, and two-stage pH control with increased production has been carried out [16,17]. The supplementation of uracil was also found to improve the curdlan fermentation yield [18]. Other decisive aspects involved in curdlan biosynthesis have been investigated, including temperature [3], carbon source [19,20], carbon concentration [3,21], nitrogen supply [5], nitrogen concentration [3,21], phosphate concentration [2,7] and oxygen supply [3,17,22,23].

Immobilized cell systems have been applied in the production of secondary metabolites and polymers. These systems possess a number of advantages, such as the repeated and prolonged use of cells, easy separation of the cells from the medium, reduced contamination risk, better operational stability and increased productivity [24,25,26,27,28,29]. Immobilization methods, including adsorption, covalent binding and entrapment in gel, have been used to provide greater stability to cells and to increase their activity; of these, the most commonly-used method is entrapment in gel [26,30]. Various matrices have been successfully employed for the immobilization of microorganisms. Among these matrices, loofa sponge is considered to be readily available, porous, low cost and biodegradable. Additionally, loofa sponge provides a simple operating technique and high stability through its continued use [25,27,28,29,31].

To the knowledge of the authors of this study, the current literature contains no studies of the immobilization of bacterial cells in any type of support for the purpose of curdlan production. Therefore, the aim of this study was to evaluate the curdlan production by *Agrobacterium* sp*.* IFO 13140 immobilized on loofa sponge, alginate and a combination of loofa sponge with alginate. The immobilization was optimized. Curdlan production was evaluated in the best immobilization matrix, and the results were compared with the production obtained by free cells.

## 2. Results and Discussion

### 2.1. Agrobacterium sp. IFO 13140 Immobilization in Different Matrices

Curdlan production by free and immobilized cells of *Agrobacterium* sp. IFO 13140 in alginate, loofa sponge and sponge-alginate showed no significant difference (*p* < 0.05), under specific immobilization conditions: 48 h of incubation, 10 mg of cells and a shaking rate of 150 rpm. There was also no significant difference in curdlan production when the assays were realized over 10 and 15 days for the same matrix. The average curdlan production was 14.87 g/L, and the yield from the total sugars (glucose) was similar for all treatments, at an average of 29.74% (Supplementary Figure S1).

Curdlan production by immobilized cells on loofa sponge, sponge-alginate and in alginate at 10 days of assay was 14.90, 13.25 and 16.52 g/L, respectively.

The loofa sponge was chosen to give continuity to this study because of its practical application and economy and because it is a renewable matrix, which fits the concept of sustainability. Furthermore, in contrast to alginate, which requires more sophisticated equipment for immobilization, involving high costs, the immobilization system in loofa sponge is more robust and can simply be done by the addition of a microbial cells suspension in the culture medium containing discs of sponge, which involve low costs and no prior chemical treatment.

Studies have shown loofa sponge to be a suitable material for cell immobilization. It is highly stable during long-term, repeated use processes. The sponge is made up of an open network of fibrous support with continuous hollow microchannels and approximately 800-mm macropores, giving it the ability to allow quick contact of immobilized cells with the surrounding medium. It is extremely light, having a specific surface area of 850 m^2^/m^−3^, a specific gravity of 0.92 g/cm^−3^, a high porosity of 79%–93%, an empty volume of 92%, a pore size of 335 ± 65 mm and a high specific pore volume of 21–29 cm^3^/g^−1^, which together show that the mass transfer in the biomatrix would be high [31].

### 2.2. The Optimization of the Agrobacterium sp. IFO 13140 Immobilization on Loofa Sponge

**Figure 1:**
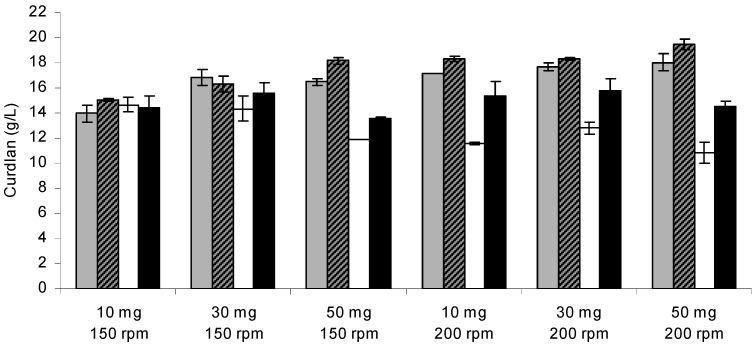
Curdlan production by *Agrobacterium* sp. IFO 13140. Free cells after 48 h (open bars) and 72 h of incubation (closed bars black); immobilized cells after 48 h (closed bars gray) and 72 h of incubation (closed bars striped). Assay conditions: 10 days of incubation in production medium at 30 °C and 150 rpm. The error bars represent standard deviations of triplicates.

Figure 1 shows the optimization of curdlan production by immobilized and free *Agrobacterium* sp. IFO 13140 cells under the interaction of different factors: shaking rate, initial biomass and incubation time. The immobilized cells of *Agrobacterium* sp. IFO 13140 showed greater curdlan production (19.45 g/L) compared to free cells (14.50 g/L), when 50 mg of cells at 200 rpm were used, and the curdlan yield from the total sugars (glucose) was 38.90% and 29%, respectively. For each amount of cell used for immobilization and for each incubation time evaluated, the curdlan production was higher with the increase in the shaking rate. This could be a sign of mass transfer, but the shaking rate could also have been responsible for the increase in the curdlan production. Other factors could be added, such as increased oxygenation.

Changes in the microenvironment caused by the matrix, as well as the easy access to and use of the substrate may have offered protection to the microorganism and provided beneficial and convenient immobilization. Previous report showed the same conclusions when loofa sponge was used as an immobilization matrix for gibberellic acid production. The authors added that the immobilization allowed for faster removal of the end products of fermentation [27]. In the ethanol production by *Saccharomyces cerevisiae* M30, the cell regeneration and protection offered by the immobilization of cells were the main factors that acted synergistically to preserve cellular activity and provide greater stability of the immobilized cell culture compared to free cell culture [29].

The regression model used to analyze the results of the optimization of the *Agrobacterium* sp. IFO 13140 immobilization in loofa sponge was adjusted properly to the experimental data, with a statistically-significant R^2^_ADJ_ of 0.82.

According to the equation obtained for the coded variables (Equation (1)), synergistic interactions were observed among the factors evaluated, demonstrating that the isolated analysis of each variable alone is not appropriate.

(1) $$\begin{array}{l} \mathrm{Curdlan}\;(g/L)=15.46+0.77\;\times \;\mathrm{incubation}\;\mathrm{time}\;+\;0.16\;\times \;\mathrm{initinal}\;\mathrm{biomass}\;+\;0.36\\ \;\times \;\mathrm{initial}\;biomass^{2}\;+\;0.35\;\times \;\mathrm{shaking}\;\mathrm{rate}\;+\;1.68\;\times \;\mathrm{cells}\;+\;0.36\;\times \;\mathrm{incubation}\;\mathrm{time}\\ \;\times \;\mathrm{shaking}\;\mathrm{rate}\;-\;0.32\;\times \;\mathrm{incubation}\;\mathrm{time}\;\times \;\mathrm{cells}\;+\;0.81\;\times \;\mathrm{incubation}\;\mathrm{biomass}\;\times \;\mathrm{cells}\\ \;+\;0.66\;\times \;\mathrm{shaking}\;\mathrm{rate}\;\times \;\mathrm{cells} \end{array}$$

**Figure 2:**
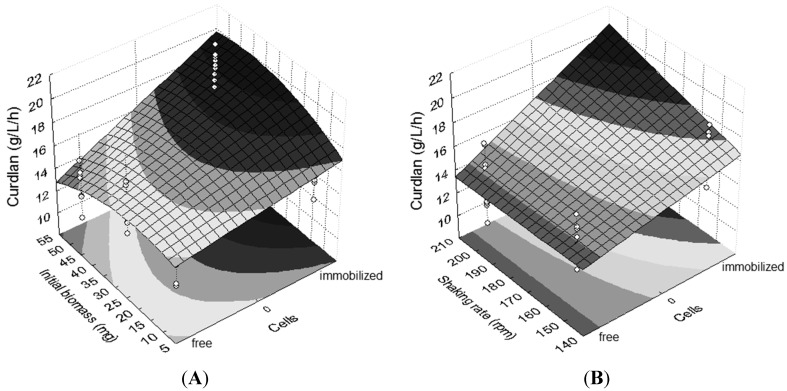
Contour plots emphasizing different effects on curdlan production. (**A**) Interaction between cells (immobilized and free) and initial biomass (10 mg, 30 mg and 50 mg); (**B**) interaction between cells (free and immobilized) and shaking rate (150 rpm and 200 rpm). Shaking rate in (A): 200 rpm. Initial biomass in (B): 50 mg. Incubation time: 72 h.

The effects of greater magnitude in curdlan production, in decreasing order, were as follows: free or immobilized cells, interaction between cells and initial biomass (Figure 2A), incubation time and interaction between cells and shaking rate (Figure 2B). Considering the regression model (Equation (1)) and the respective contour surfaces (Figure 2), it is suggested that the best immobilization conditions were 72 h of incubation, 50 mg of cells and a shaking rate of 200 rpm.

Based on the regression model used, it was found that the process of *Agrobacterium* sp. IFO 13140 immobilization significantly increased the production in 17.27% of curdlan. The ε-poly-l-lysine production by *Kitasatospora* spp. MY 5–36 immobilized on loofa sponge was 1.35-times greater than the production by free cells [32].

The immobilization of *Agrobacterium* sp. IFO 13140 for 72 h showed better results compared to those for 48 h and resulted in an increase of 7.92% in curdlan production.

The maintenance of immobilized cells in the shaking rate at 200 rpm promoted an increase of 3.65% in curdlan production by *Agrobacterium* sp. IFO 13140. An increase in curdlan production of 76.96% by free cells of *Rhizobium radiobacter* ATCC 6466 was achieved with an increase in the shaking rate from 120 to 180 rpm [3].

In contrast to what was observed for the immobilized cells, a reduced initial biomass resulted in higher curdlan production by free cells (Figure 1). An increase in curdlan production of 7.94% was achieved with a decrease in the cell concentrations from 0.50 to 0.30 g/L. A high density of free cells may cause problems of mass transference in the culture medium and lead to cellular lysis by increasing the concentration of nitrogen in the medium, thereby stimulating cell proliferation and reducing curdlan synthesis rates. Furthermore, high cell concentrations can lead to high oxygen demand and higher energy requirements for cell maintenance, so that low curdlan production may occur. An amount of 60 g/L of curdlan was obtained when using a cell concentration produced of 16 g/L, whereas a cell concentration produced of 38 g/L resulted in a production of 25 g/L [19]. A reduction in the production of 5.50 g curdlan/g cells to 1.70 g curdlan/g cells was also obtained by using a high cell density (21 g cell/L) [22].

### 2.3. The Influence of Shaking Rate on Curdlan Production

The 150-rpm shaking rate was more favorable for curdlan production, both by immobilized cells (19.45 g/L) compared to the shaking rates of 110 (11.77 g/L) and 200 rpm (14.60 g/L) and by free cells (14.12 g/L). Control of the shaking rate is an important factor for curdlan production, because suboptimal shaking may not provide adequate oxygen supply for an increase in biomass [17], whereas too much shaking can lead to a stressful environment for a microbial cell [3] (Supplementary Figure S2).

The yield at 150 rpm was 38.90% for immobilized cells and 28.24% for free cells. There was no statistically-significant difference between the curdlan productions by free and immobilized cells at 200 rpm, which were 15.35 and 14.60 g/L, respectively. For the other treatments, the immobilization provided higher production.

The shaking rate of 110 rpm provided an unfavorable environment for curdlan production by free cells (approximately 0.58 g/L), possibly due to insufficient aeration. The dissolved oxygen concentration in the production medium is one of the most important factors in curdlan production. When *Agrobacterium* sp. ATCC 31749 was submitted to various dissolved oxygen levels in a culture environment, changes occurred in the gene transcription levels involved in the tricarboxylic acid cycle (*icd*, *sdh B* and *mdh*), in lipopolysaccharide/peptide-glucan biosynthesis (*glm M*), in the curdlan production route (*gal U*) and in the electron transfer chain (*cyo A*, *cat D*, and *fix N*). The translation rates under 50% dissolved oxygen were higher than obtained in 30%, where four of the eight analyzed genes showed no significant change and curdlan production was reduced by approximately 25%. With 75% dissolved oxygen, all genes were repressed, and curdlan production was reduced by half compared to that obtained under 50% oxygen, which showed that gene transcription levels are determined by the concentration of oxygen and not simply its presence or absence [22].

### 2.4. Study of Curdlan Production Kinetics

**Figure 3:**
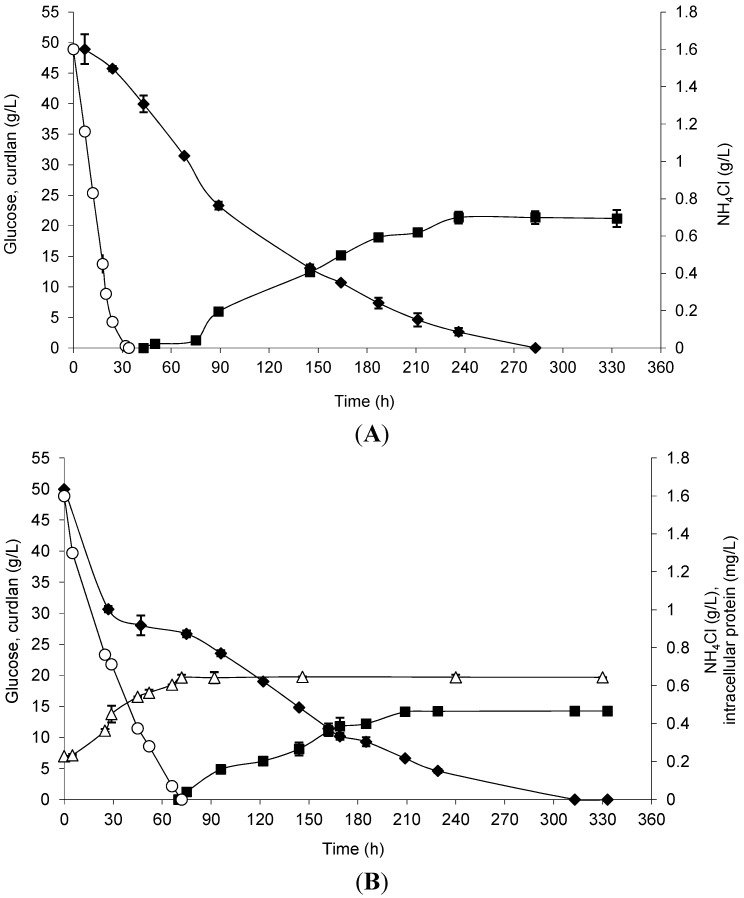
Curdlan production (closed squares), biomass represented by intracellular protein (open triangles), glucose consumption (closed diamonds) and NH_4_Cl (open circles) by *Agrobacterium* sp. IFO 13140 immobilized on loofa sponge (**A**) and free cells (**B**) at 30 °C and 150 rpm for 14 days of incubation. The immobilization conditions used were 72 h of incubation, 50 mg of cells and a shaking rate of 200 rpm. The error bars represent standard deviations of triplicates.

Curdlan production by free and immobilized cells was evaluated considering the previously optimized conditions, and the results are shown in Figure 3. Curdlan production by immobilized cells started 34 h earlier than that observed in free cells. Glucose exhaustion and maximum curdlan production occurred at approximately 10 days in the assay. The total glucose consumption was 94.74% by immobilized cells and 90.82% by free cells, and the maximum curdlan production was 21.35 and 14.20 g/L, respectively. In Figure 3A, it is possible to note the use of ammonium chloride until 30 h of incubation time, which means that the immobilized cells grew in the loofa sponge.

As previously described, *Alcaligenes faecalis* WX-C12 consumed glucose for cell maintenance and metabolic energy supply for curdlan biosynthesis [33]. Therefore, the maximum curdlan production can be considered as a function of the specific rate of glucose consumption and significantly varies with the conditions of cultivation.

**Figure 4:**
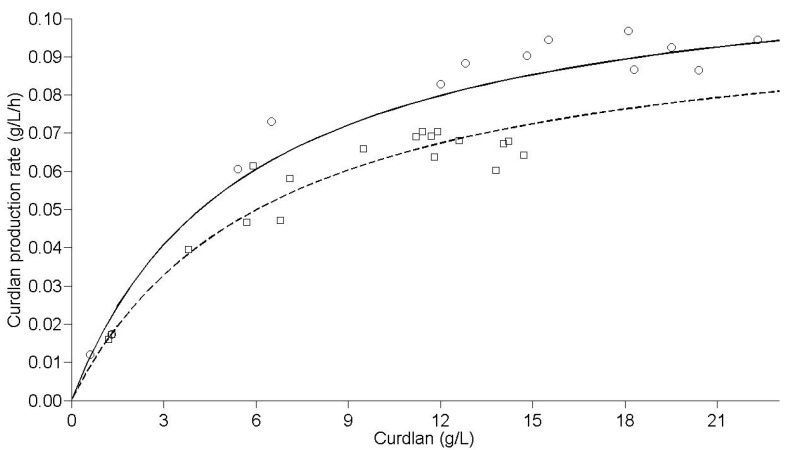
Curdlan production rate by *Agrobacterium* sp. IFO 13140. Predicted rate (solid line) and observed rate (open circles) for immobilized cells and predicted rate (sectioned line) and observed rate (open squares) for free cells, at 30 °C and 150 rpm.

A proper fit to the experimental data was achieved in relation to the data predicted using the kinetic model, with an R^2^_ADJ_ of 0.97 for immobilized cells and 0.99 for free cells. A hypothesis test (*t*-test) for the parameters 1/k_0_ and 1/k_1_ was applied, and a statistically-significant difference was found at 1% for both parameters regarding the use of free or immobilized cells. The speed constants were higher for immobilized cells (k_0_ = 1.17 × 10^−1^ g/L/h and k_1_ = 2.08 × 10^−2^ h^−1^) compared to free cells (k_0_ = 1.04 × 10^−1^ g/L/h and k_1_ = 1.60 × 10^−2^ h^−1^). Therefore, the immobilization process resulted in a higher maximum production speed, and this speed was reached in a shorter time (Figure 4). This setting has confirmed the superiority of the immobilization system of *Agrobacterium* sp. IFO 13140 in relation to free cells, under the immobilization conditions and production used.

### 2.5. Operational Stability

**Figure 5:**
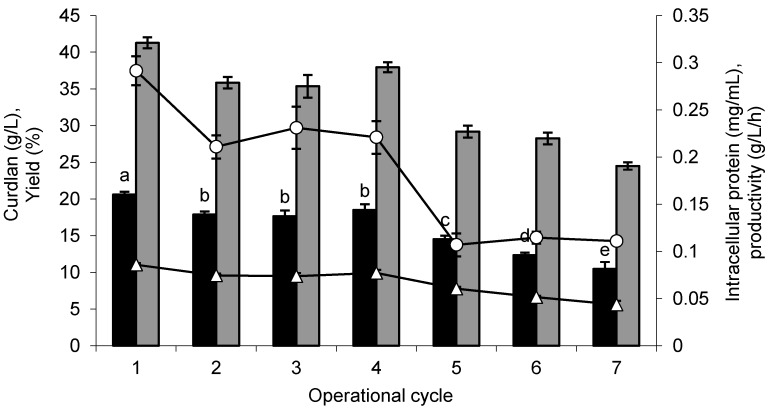
Curdlan production by *Agrobacterium* sp. IFO 13140 immobilized in loofa sponge (closed bars black), yield (closed bars gray), volumetric productivity (open triangles) and suspended biomass (open circles), in seven consecutive cycles of 10 days each at 30 °C and 150 rpm. The error bars represent standard deviations of triplicates.

For operational stability, the conditions previously optimized for immobilization and curdlan production were used. The curdlan production by immobilized cells was evaluated by seven repeated cycles of 10 days each. The glucose consumption was 99.37% in the first five cycles and dropped to 87.36% and 85.40% in the sixth and seventh cycles, respectively, and the increased consumption led to increased production levels. In the first cycle, the curdlan production was 20.62 g/L, 31.62% higher than that obtained by free cells (14.10 g/L) and higher than those obtained by the immobilized cells in the other operational cycles (Figure 5).

Until the fourth cycle, the immobilized cells maintained an average of 87.40% of the curdlan production obtained in the first cycle and showed a reduction of 29.43%, 40.10% and 49.32% only in the fifth, sixth and seventh cycles, respectively. Considering that the production of curdlan occurred for several consecutive cycles, it is possible to suppose that the immobilization matrix did not negatively affect the mass transfer.

Considering that the curdlan serves as an energy reserve, its production by free cells in the second cycle was null, due to the presence of curdlan in the medium since the beginning of the second cycle, when the contents of curdlan present in the first cycle were transferred with the cells for the second cycle. The glucose consumption was 99% in the first cycle and 79% in the second cycle. Curdlan is used as both an energy reserve and a means for cellular adherence to solid surfaces and to improve cell survival [34]. Other authors have also obtained good operational stability through the use of loofa sponge as a matrix for immobilization. Ethanol production was obtained by *Saccharomyces cerevisiae* in the course of four cycles with less than a 5% reduction in production [25]. The decreased production of ε-poly-l-lysine by *Kitasatospora* sp. MY 5–36 was detected only after the fourth production cycle [32].

In Figure 5, it is also observed that the highest volumetric productivity of curdlan achieved was 0.09 g/L/h, which was obtained in the first cycle; thereafter, a decrease occurred throughout the cycle, reaching 0.04 g/L/h in the last cycle. The volumetric productivity obtained by free cells was 0.06 g/L/h of curdlan in the first cycle. Other authors working only with free cells, large volumes of culture medium and incubation times of approximately 120 h obtained different productivity values. The use of the *Agrobacterium* sp. ATCC 31750 mutant afforded a volumetric productivity of 0.63 g/L/h of curdlan [15]. When a parental strain was used, 0.55 g/L/h of curdlan was obtained with the addition of uracil 46 h after ammonia depletion [18] and 0.20 g/L/h of curdlan when sucrose was utilized as a carbon source [19]. A volumetric productivity of 0.36 g/L/h of curdlan by *Agrobacterium* sp. ATCC 31749 was reached when a rate of dissolved oxygen equal to 60% was utilized [23].

The determination of intracellular protein confirmed the cell desorption from the immobilization matrix. These cells were not transferred to the subsequent production cycle. The average protein content in the first four cycles was 0.24 mg/mL, and in the last three cycles, there was an average reduction of 53.53% (Figure 5). The formation of free biomass in all cycles evaluated contributed to glucose consumption and curdlan formation. The increased number of dead cells immobilized due to its reuse may also have contributed to the decreased production of curdlan after the fourth cycle, which was described previously for the biosynthesis of ε-poly-l-lysine [32] and gibberellic acid [27], by microorganisms immobilized on loofa sponge.

The percentage of biomass present in the matrix after immobilization was 21.80%, in relation to the initial biomass used. The nitrogen consumption in the production medium allowed the growth of the biomass in the loofa sponge. This biomass was partially desorbed, resulting in 10.20 mg of the biomass attached to loofa sponge, and 13 mg of the biomass desorbed after the first production cycle of curdlan. The free biomass increased 3.30-times compared with the initial biomass used after the first production cycle. Therefore, the specific rate of production obtained in the immobilization system (immobilized and desorbed cells) was 0.37 g curdlan/g cells/h and in the cell free system was 0.03 g curdlan/g cells/h. The highest production of curdlan by immobilized cells is due to better biocatalytic activity provided by the system. It seems likely that the lower specific rate of production obtained by free cells resulted from a higher energy requirement for cellular maintenance and a higher oxygen demand.

### 2.6. Storage Stability

Because immobilized microbial cells have the advantage of a long period of use, the storage stability of *Agrobacterium* sp. IFO 13140 cells immobilized on loofa sponge and kept at 4 *°*C for 300 days was evaluated. Curdlan production remained constant over time, with an average value of 13.05 g/L, and no statistically-significant difference was observed compared to that obtained without storing cells (13.95 g/L). Therefore, immobilized cells retained their metabolic activity during the storage periods investigated (60, 120, 180, 240 and 300 days), demonstrating a high storage stability. The average yield was 26.10%.

### 2.7. Curdlan Characterization

In the present study, Fourier transform infrared (FTIR) spectroscopy was carried out, and the obtained spectrum showed band transmittance at 3410, 2928, 1373, 1317, 1160 and 1080 cm^−1^, which, respectively, correspond to the bonds -OH, -CH_2_, CH, CH_2_, C_1_-O-C_3_ and C-O [20,21]. The bands observed for the commercial curdlan were presented in accordance with the bands of the curdlan produced by *Agrobacterium* sp. IFO 13140 (Supplementary Figure S3). Therefore, the curdlan produced by immobilized cells is formed exclusively of glucose. The FTIR spectrum also showed an absorption band at 890 cm^−1^, indicating that d-glucopyranose has (1→3)-β-glycosidic bonds. The β-configuration was also verified by the natural gums produced by strains of *Agrobacterium* sp. ATCC 31750 [15] and *Rhizobium radiobacter* ATCC 6466 [3].

## 3. Experimental Section

The strain *Agrobacterium* sp. IFO13140, previously identified as *Alcaligenes faecalis*, was acquired from the Institute for Fermentation of Osaka (Osaka, Japan). The loofa sponge plant was purchased in a local market (Maringa, PR, Brazil). The other chemicals used were of analytical grade and from Sigma-Aldrich (St. Louis, MO, USA) or Merck (Darmstadt, Germany).

### 3.1. Microorganism and Growth Conditions

The reactivation of the *Agrobacterium* sp. IFO13140 was conducted in the growth medium indicated by the supplier (g/L): polypeptone (10), yeast extract (2) and MgSO_4_·7H_2_O (1), pH 7. The microorganism was grown at 30 °C under shaking at 120 rpm for 48 h. After its lyophilization, the microorganism was stored in a freezer (−18 °C).

To obtain the pre-inoculum, cellular reactivation was conducted in a 250-mL Erlenmeyer flask containing 10 mg of lyophilized cells and 100 mL of liquid medium (g/L): sucrose (20), yeast extract (6) and MgSO_4_·7H_2_O (1), pH 7. The flasks were incubated at 30 °C and 150 rpm for 48 h.

The reactivation of lyophilized cells of *Agrobacterium* sp. IFO 13140 was evaluated in triplicate using 30 mg of the microorganism and medium under the conditions used for the pre-inoculum. After the growth of the microorganism, the absorbance measurements were made at 560 nm, and the reactivation was efficient. The amount of cells reactivated and calculated by dry weight was 1.29 g/L.

Curdlan production was performed in a 250-mL Erlenmeyer flask containing 100 mL of liquid medium [34] (g/L): glucose (50), KH_2_PO_4_ (2.70), NH_4_Cl (1.60), MgSO_4_ (0.50) and CaCO_3_ (3). Trace elements were also added to the medium (10 mL/L). The composition of trace elements (g) in 1 L of 0.1 mol/L HCl was: FeCl_3_·6H_2_O (1), MnCl_2_·4H_2_O (1), CaCl_2_ (1) and ZnCl (1). The pH was maintained between 6.50 and 7 during curdlan production.

### 3.2. Agrobacterium sp. IFO 13140 Immobilization in Different Matrices

Loofa sponge (*Luffa cylindrica*), alginate and a matrix derived from the combination of alginate and loofa sponge (sponge-alginate) were used for the immobilization of bacterial cells. For the immobilization on loofa sponge, the seedless dried fruit of *Luffa cylindrical* was used. The loofa sponges were standardized (24 mm diameter and 2–4 mm thick, weighing 0.13 g) and treated as in a previous report [28]. Three disks of loofa sponge were added to a 250-mL Erlenmeyer flask with 100 mL of pre-inoculum medium and 10 mg of the lyophilized inoculum. The inocula were kept at 30 °C and 150 rpm for 48 h and were subsequently transferred to curdlan production medium.

The immobilization in alginate was performed according to a previous report [30], with several modifications. A solution of 3% sodium alginate (*w*/*v*) was prepared by dissolving sodium alginate in a solution of 0.90% NaCl (*w*/*v*). Ten milligrams of lyophilized cells were cultured for 48 h at 30 °C and 150 rpm. Subsequently, the cells were removed from the pre-inoculum medium by centrifugation at 2800× *g* for 10 min (Model Rotanta 460 R, Andreas Hettich GmbH & Co. KG, Tuttlingen, Germany), resuspended in 0.90% NaCl (*w*/*v*) and added to the 3% sodium alginate solution (*w*/*v*). The alginate-cell solution was dripped into a 1.47% CaCl_2_ solution, and the beads obtained were washed in 0.90% NaCl (*w*/*v*) and transferred to curdlan production medium. The mean diameter of the beads was 3 mm.

The immobilization matrix sponge-alginate was performed as previously described [28], with several modifications. To prepare the matrix, three discs of sponge were immersed in the alginate-cell solution obtained as described above, transferred to a 1.47% CaCl_2_ (*w*/*v*) solution and kept at 70 rpm for 15 min. Thereafter, the matrix was washed in 0.90% NaCl (*w*/*v*) and transferred to curdlan production medium.

The curdlan production by free cells was evaluated. Ten milligrams of lyophilized cells were cultured for 48 h at 30 °C and 150 rpm. Subsequently, the free cells were centrifuged at 2800× *g* for 10 min, washed in 0.90% NaCl (*w*/*v*) and transferred to curdlan production medium. All treatments were kept at 30 °C and 150 rpm for 15 days.

### 3.3. The Optimization of Agrobacterium sp. IFO 13140 Immobilization on Loofa Sponge

*Agrobacterium* sp. IFO 13140 immobilization on loofa sponge was optimized by evaluating the combination of three variables: time of incubation (48 and 72 h), initial biomass (10, 30 and 50 mg) and shaking rate (150 and 200 rpm). The experiments were performed according to a factorial design of mixed levels (2^3^ × 3^1^) [35] (Supplementary Table S1). The treatments were conducted in the pre-inoculum medium, which was maintained at 30 °C. The same procedures were applied to free cells. After incubation periods for immobilization, the loofa sponges were transferred to curdlan production medium and maintained at 30 °C and 150 rpm for 10 days. The free cells were centrifuged at 2800× *g* for 10 min, washed in 0.90% NaCl (*w*/*v*) and transferred to production medium.

### 3.4. The Influence of Shaking Rate on Curdlan Production

After the immobilization period under optimal conditions previously established, the loofa sponges were transferred to curdlan production medium and maintained at 30 °C for 10 days with a shaking rate of 110, 150 or 200 rpm. The free cells, after growth in the pre-inoculum medium, were centrifuged at 2800× *g* for 10 min, washed in 0.90% NaCl (*w*/*v*) and transferred to production medium, which was kept under the same conditions used for immobilized cells.

### 3.5. Kinetic Studies of Curdlan Production

Based on the knowledge of the optimal conditions for *Agrobacterium* sp. IFO 13140 immobilization and curdlan production, an assay was performed over 14 days using the optimized parameters, and glucose and nitrogen consumption were evaluated, as was the production of curdlan and biomass. In this assay, to describe the production speed of the curdlan, by either immobilized cells or free cells, a kinetic model of an order between 0 and 1 was used, as described in Equation (2) [36]:

(2) $$\mathrm{Speed}\;(g/L/h)=\frac{k_{0}\;k_{1}\;\left[C\right]}{k_{0}\;+\;k_{1}\;\left[C\right]}$$

where [C] is the curdlan concentration (g/L) and k_0_ and k_1_ are, respectively, the speed kinetic constants of order zero and order one. This kinetic model is suitable to describe processes that have an initial stage in which the speed is proportional to the monitored species concentration (order one) and a final stage that is independent of concentration (zero order). The speed kinetic constants can be calculated using the method of least squares, where Equation 2 is in a linear form (Equation (3)):

(3) $$\frac{1}{v}=\frac{1}{k_{0}}\;+\;\frac{1}{k_{1}}\;\times \;\frac{1}{\left[C\right]}$$

### 3.6. Operational Stability

The optimal conditions for the immobilization of *Agrobacterium* sp. IFO 13140 and curdlan production were applied in this experiment for free and immobilized cells. The curdlan production was evaluated in consecutive cycles using immobilized and free cells of *Agrobacterium* sp. IFO13140. Cycles were performed over 10 days, and at the end of each cycle, the loofa sponges containing immobilized cells were removed from the medium and transferred to a new production medium. The free cells were centrifuged at 2800× *g* for 15 min, washed in 0.90% NaCl (*w*/*v*) and transferred to a new medium. The biomass present in the production medium was evaluated at the end of each cycle.

### 3.7. Storage Stability

To assess the storage stability, 10 mg of *Agrobacterium* sp. IFO 13140 cells were immobilized in loofa sponge for 48 h at 150 rpm. Subsequently, the cells were stored at 4 °C for 300 days. At 60, 120, 180, 240 and 300 days, immobilized cells were transferred to the production medium and maintained at 30 °C and 150 rpm for 10 days.

### 3.8. Analytical Methods

#### 3.8.1. Curdlan Quantification and Characterization

To recover and quantify the curdlan produced by *Agrobacterium* sp. IFO 13140, a previously reported method [2] was used with modifications. One milliliter of sample was added to 15 mL of a 3 mol/L NaOH solution and incubated at room temperature for 30 min to solubilize the curdlan. Subsequently, the mixture was centrifuged at 9072× *g* for 15 min at 4 °C, and the curdlan present in the supernatant was precipitated by adding 3 mol/L HCl until the solution reached a pH of 6.50. The recovery of the precipitated curdlan was performed with centrifugation at 9072× *g* for 15 min at 4 °C, and the product was washed three times with distilled water to remove salts. The amount of curdlan was determined by weighing after drying at 80 °C to a constant weight.

FTIR spectroscopic analysis of curdlan was performed using the KBr disc method. Approximately 200 mg of dry KBr and 2 mg curdlan were mixed and ground to a fine powder. The pellet was placed in the FTIR spectrophotometer holder (Model Vertex 70x, Bruker, Billerica, MA, EUA), and the transmittance was measured from 400 to 4000 cm^−1^ at a resolution of 4 cm^−1^. The values obtained for the curdlan sample of the present study were compared with those of an authentic curdlan sample (obtained from Wako, Richmond, VA, EUA).

#### 3.8.2. The Determination of Glucose and Nitrogen Concentration

The glucose consumption was monitored using the enzymatic colorimetric test (Glucose Bioliquid Kit, obtained from Laborclin, Pinhais, PR, Brazil). The nitrogen consumption was monitored using the indophenol blue method [37].

#### 3.8.3. Biomass Determination

The best immobilization and curdlan production conditions were used to determine the biomass immobilized in the matrix and the biomass present in loofa sponge after the first production cycle of curdlan. Three discs of loofa sponge were weighed before and after incubation in the pre-inoculum medium. Prior to weighing, the sponges were dried for 24 h at 80 °C. After one production cycle of curdlan, the sponges were washed with 0.50 mol/L NaOH solution to remove the microbial cells and curdlan. The solution was maintained to stand for 30 min to solubilize the curdlan. The recovery of the biomass was performed with centrifugation at 9072× *g* for15 min at 4 °C, and the pellet was washed with distilled water. The amount of biomass was determined by weighing after drying at 80 °C.

The biomass of free cells was determined after the first production cycle of curdlan under the same conditions used for immobilized cells. Curdlan was solubilized with 0.50 mol/L NaOH solution and the biomass determined according to the methodology mentioned above.

During the kinetic studies of curdlan production, the biomass was determined by detecting the intracellular protein concentration [38]. The desorbed biomass was monitored by the same methodology during the operational stability assay. One milliliter of 1 mol/L NaOH was added to 1 mL of sample, and the solution obtained was boiled for 20 min and centrifuged for 5 min at 2800× *g*. To the supernatant, 3 mL of Bradford reagent were added. Absorbance readings were then conducted at 595 nm in a spectrophotometer.

### 3.9. Statistical Analysis

All assays were conducted in triplicate for each treatment, and the efficiency was obtained by monitoring the average of two replicates for the curdlan production.

The results were submitted to analysis of variance (ANOVA) and Tukey tests at a 5% significance level, except those obtained for test optimization of cell immobilization. The program Statistica 7.0/2007 (Stat Soft, Inc., Tulsa, OK, USA) was used to analyze all of the data obtained.

## 4. Conclusions

*Agrobacterium* sp. IFO 13140 immobilization on loofa sponge was innovative, and the experimental design of mixed levels allowed for the determination of the optimum cell amount, incubation time and stirring rate. The continuous production of curdlan by immobilized cells occurred by seven successive cycles and at a rate 31.62% higher than that for free cells. Storage stability without a decrease in production occurred during 300 days of storage. The innovative use of loofa sponge, an environmentally-friendly matrix, inexpensive, strong, resistant to operating conditions and with an open structure, gave good performance for curdlan production, thus making the process potentially suitable for industrial scale-up. Studies on scale-up must be performed to assess the economic viability of the production process.

## Supplementary Materials

Supplementary materials can be accessed at: http://www.mdpi.com/1420-3049/20/05/7957/s1.
