# Supplementary material for: Effective Immobilization of Agrobacterium sp. IFO 13140 Cells in Loofa Sponge for Curdlan Biosynthesis
Source: Molecules. 2015 May 4;20(5):7957–73. doi: 10.3390/molecules20057957 (PMC6272340; doi:10.3390/molecules20057957)
Supplement: Supplementary file 1 [file molecules-20-07957-s001.pdf]

Supplementary Materials

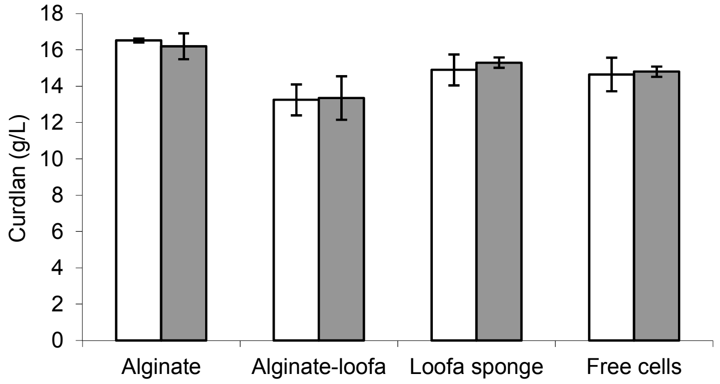

Figure S1. Curdlan production by *Agrobacterium* sp. IFO 13140 immobilized in alginate, sponge-alginate loofa sponge and free cells at 30 °C and 150 rpm, at 10 days (open squares) and 15 days (closed squares gray). The immobilization conditions were 48 h of incubation, 10 mg of cells and a shaking rate of 150 rpm.

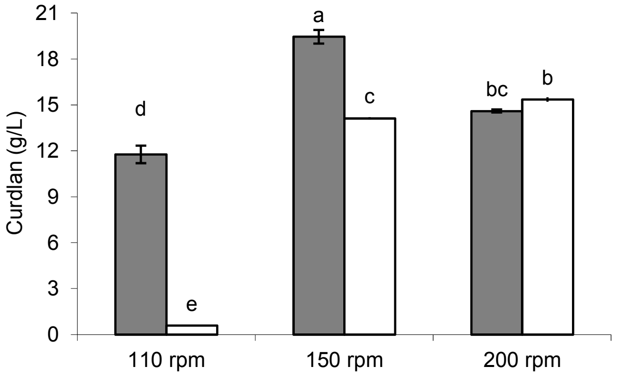

Figure S2. Curdlan production by *Agrobacterium* sp. IFO 13140: free cells (open squares) and immobilized cells (closed squares gray). Assay conditions: 10 days of incubation in production medium at 30 °C and under different agitation speeds (rpm). Different letters in different columns represent statistically-significant differences ($p < 0.05$).

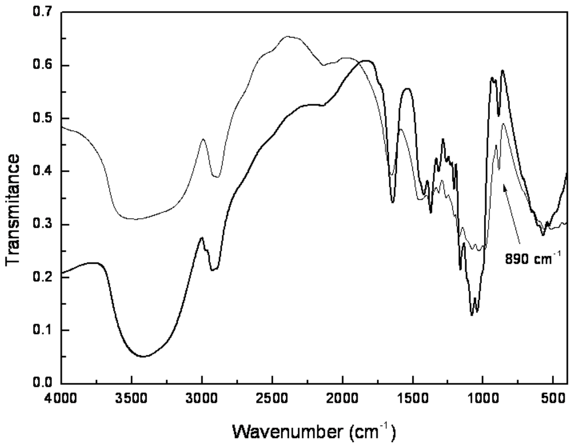

Figure S3. FTIR spectra of curdlan from *Agrobacterium* sp. IFO 13140 (thick line) and commercial curdlan (thin line).

Table S1. Factors and levels of the asymmetric factorial design $2^3 \times 3^1$ used for the test optimization of *Agrobacterium* sp. IFO 13140 immobilization.

Experiment	Incubation Time (h)	Initial Biomass (mg)	Shaking Rate (rpm)	Cells	Curdlan (g/L)
1	48 (−1)	10 (−1)	150 (−1)	Free (−1)	15.3
2	48 (−1)	10 (−1)	150 (−1)	Free (−1)	14
3	72 (1)	10 (−1)	150 (−1)	Free (−1)	13.4
4	72 (1)	10 (−1)	150 (−1)	Free (−1)	15.5
5	48 (−1)	50 (1)	150 (−1)	Free (−1)	11.9
6	48 (−1)	50 (1)	150 (−1)	Free (−1)	11.9
7	72 (1)	50 (1)	150 (−1)	Free (−1)	13.7
8	72 (1)	50 (1)	150 (−1)	Free (−1)	13.5
9	48 (−1)	10 (−1)	200 (1)	Free (−1)	11.5
10	48 (−1)	10 (−1)	200 (1)	Free (−1)	11.7
11	72 (1)	10 (−1)	200 (1)	Free (−1)	14
12	72 (1)	10 (−1)	200 (1)	Free (−1)	16.7
13	48 (−1)	50 (1)	200 (1)	Free (−1)	11.8
14	48 (−1)	50 (1)	200 (1)	Free (−1)	9.9
15	72 (1)	50 (1)	200 (1)	Free (−1)	15
16	72 (1)	50 (1)	200 (1)	Free (−1)	14
17	48 (−1)	10 (−1)	150 (−1)	immobilized (1)	13.2
18	48 (−1)	10 (−1)	150 (−1)	immobilized (1)	14.7
19	72 (1)	10 (−1)	150 (−1)	immobilized (1)	15.15
20	72 (1)	10 (−1)	150 (−1)	immobilized (1)	14.9
21	48 (−1)	50 (1)	150 (−1)	immobilized (1)	16.8
22	48 (−1)	50 (1)	150 (−1)	immobilized (1)	16.2
23	72 (1)	50 (1)	150 (−1)	immobilized (1)	17.85
24	72 (1)	50 (1)	150 (−1)	immobilized (1)	18.5
25	48 (−1)	10 (−1)	200 (1)	immobilized (1)	17.1
26	48 (−1)	10 (−1)	200 (1)	immobilized (1)	17.2
27	72 (1)	10 (−1)	200 (1)	immobilized (1)	18.5
28	72 (1)	10 (−1)	200 (1)	immobilized (1)	18.1
29	48 (−1)	50 (1)	200 (1)	immobilized (1)	17.3
30	48 (−1)	50 (1)	200 (1)	immobilized (1)	18.8
31	72 (1)	50 (1)	200 (1)	immobilized (1)	19.9
32	72 (1)	50 (1)	200 (1)	immobilized (1)	19
33	48 (−1)	30 (0)	150 (−1)	Free (−1)	15.5
34	72 (1)	30 (0)	150 (−1)	Free (−1)	16.5
35	48 (−1)	30 (0)	200 (1)	Free (−1)	13.3
36	72 (1)	30 (0)	200 (1)	Free (−1)	16.8
37	48 (−1)	30 (0)	150 (−1)	immobilized (1)	16.1
38	72 (1)	30 (0)	150 (−1)	immobilized (1)	15.6
39	48 (−1)	30 (0)	200 (1)	immobilized (1)	17.35
40	72 (1)	30 (0)	200 (1)	immobilized (1)	18.2
41	48 (−1)	30 (0)	150 (−1)	Free (−1)	13.2
42	72 (1)	30 (0)	150 (−1)	Free (−1)	14.6
43	48 (−1)	30 (0)	200 (1)	Free (−1)	12.3
44	72 (1)	30 (0)	200 (1)	Free (−1)	14,7
45	48 (−1)	30 (0)	150 (−1)	immobilized (1)	17.55
46	72 (1)	30 (0)	150 (−1)	immobilized (1)	17.05
47	48 (−1)	30 (0)	200 (1)	immobilized (1)	18
48	72 (1)	30 (0)	200 (1)	immobilized (1)	18.4
